# Draft Genome Sequence of *Marinomonas fungiae* Strain AN44^T^ (JCM 18476^T^), Isolated from the Coral *Fungia echinata* from the Andaman Sea

**DOI:** 10.1128/genomeA.00112-18

**Published:** 2018-03-08

**Authors:** Prabla Kumari, Jhasketan Badhai, Subrata K. Das

**Affiliations:** a Department of Biotechnology, Institute of Life Sciences, Bhubaneswar, Odisha, India

## Abstract

Marinomonas fungiae strain AN44^T^ was isolated from mucus of the coral Fungia echinata. Optimum growth occurs at 3 to 5% NaCl. The draft genome is 4.2 Mb, with 3,776 protein-coding genes. It harbors genes for the degradation of aromatic compounds, such as quinate, ferulate, *p*-coumarate, protocatechuate, and *p*-hydroxyphenylacetate.

## GENOME ANNOUNCEMENT

Marinomonas fungiae strain AN44^T^ (JCM 18476^T^) is an aerobic, Gram-negative, motile, and rod-shaped bacterium belonging to the class Gammaproteobacteria and was isolated from mucus of the coral Fungia echinata from the Andaman Sea (1). The draft genome sequence will help in understanding the plasticity and diversity of metabolic pathways for the degradation of aromatic compounds/pollutants in marine bacteria. Further, the strain harbors two unique mobile genetic elements belonging to the SXT integrating conjugative element (ICE) family (2).

The draft genome sequence of M. fungiae strain AN44^T^ was generated at the DOE Joint Genome Institute (JGI) using the Illumina HiSeq 2000 platform (3). An Illumina standard shotgun library was constructed and sequenced using the Illumina HiSeq 2000 platform, which generated 10,961,766 reads totaling 1,655.2 Mb. The filtered Illumina reads were assembled using the Velvet (4), wgsim (https://github.com/lh3/wgsim), and AllPaths-LG (5) tools. The final draft assembly contains 42 contigs in 36 scaffolds, totaling 4.2 Mb, with an input read coverage of 268.9-fold. The largest and *N*_50_ contigs are 554.8 kb and 268.5 kb, respectively, with a G+C content of 46.2%.

The genome was annotated using the JGI Microbial Genome Annotation Pipeline (6). Genes were identified using the Prodigal (7) and GenePRIMP (8) programs. The predicted coding sequences (CDSs) were translated and used to search the National Center for Biotechnology Information (NCBI) nonredundant, UniProt, TIGRFAM, Pfam, KEGG, COG, and InterPro databases. The tRNAs, rRNAs, and other noncoding RNA genes were identified by searching the genome using the tRNAscan-SE tool (9), rRNA gene models built from SILVA (10), and Infernal (http://infernal.janelia.org), respectively. The draft genome contains a total of 3,776 CDSs, 78 pseudogenes, 53 tRNAs, 16 rRNAs (5 5S rRNAs, 9 16S rRNAs, and 2 23S rRNAs), 5 noncoding RNAs (ncRNAs), and 1 clustered regularly interspaced short palindromic repeat (CRISPR). Based on COG functional categories, the CDSs of the M. fungiae genome were distributed into categories of amino acid transport and metabolism (9.84%), carbohydrate transport and metabolism (5.92%), cell cycle control, cell division, and chromosome partitioning (1.08%), cell motility (3.51%), cell wall/membrane/envelope biogenesis (5.06%), chromatin structure and dynamics (0.09%), coenzyme transport and metabolism (5.47%), defense mechanisms (2.18%), energy production and conversion (6.61%), extracellular structures (0.44%), function unknown (5.41%), general function prediction only (7.31%), inorganic ion transport and metabolism (5.19%), intracellular trafficking, secretion, and vesicular transport (1.55%), lipid transport and metabolism (3.35%), mobilome prophages and transposons (1.08%), nucleotide transport and metabolism (2.47%), posttranslational modification, protein turnover, and chaperones (4.15%), RNA processing and modification (0.03%), replication, recombination, and repair (3.83%), secondary metabolite biosynthesis, transport, and catabolism (2.12%), signal transduction mechanisms (8.13%), transcription (7.97%), translation, ribosomal structure, and biogenesis (7.18%), and unassigned (28.88%).

Further, putative genes or gene clusters for peripheral aromatic compound degradation pathways of quinate, ferulate, and *p*-coumarate, along with a single operonic gene cluster for central aromatic compound degradation via the protocatechuate *meta*-cleavage pathway, were found in the genome. Additionally, a single operonic gene cluster for the degradation of *p*-hydroxyphenylacetate was found.

### Accession number(s).

This whole-genome shotgun project has been deposited at DDBJ/EMBL/GenBank under the accession number LIQF00000000. The version described in this paper is the first version, LIQF01000000, and consists of sequences LIQF01000001 to LIQF01000036.
